# Sarcopenia and Skeletal Muscle Dysfunction in Liver Cirrhosis: Clinical Perspectives of Myostatin Inhibition

**DOI:** 10.3390/biomedicines14092082

**Published:** 2026-09-16

**Authors:** Olga Sukocheva, Damian Harding, Tsai-Wing Ow, Edmund Tse

**Affiliations:** 1 Department of Gastroenterology and Hepatology, Royal Adelaide Hospital, CALHN, Port Rd, Adelaide, SA 5000, Australia; damian.harding@adelaide.edu.au (D.H.);; 2 Faculty of Health and Medical Sciences, Adelaide University, Adelaide, SA 5000, Australia; 3 Department of Gastroenterology and Hepatology, Lyell McEwin Hospital, Elizabeth Vale, SA 5112, Australia

**Keywords:** cirrhosis, gene therapy, mTOR, myostatin, myokines, sarcopenia, testosterone

## Abstract

Liver cirrhosis is frequently complicated by sarcopenia, a progressive and systemic loss of skeletal muscle mass and function that contributes substantially to increased morbidity, mortality, and healthcare utilisation. The pathogenesis of sarcopenia in cirrhosis is multifactorial, involving hyperammonaemia, altered protein turnover, hormonal and cytokine dysregulation, chronic inflammation, malnutrition, and broader metabolic disturbances. Increasing evidence suggests that these convergent pathological processes are mediated, at least in part, through dysregulation of myokine signalling pathways, particularly those involving myostatin. Myostatin, in concert with downstream effectors such as mechanistic target of rapamycin (mTOR), plays a central role not only in skeletal muscle homeostasis but also in systemic metabolic regulation, highlighting its relevance beyond muscle biology alone. Therapeutic strategies targeting myostatin signalling have been developed, with several myostatin inhibitors advancing into clinical trials. Emerging data indicate that these agents may increase skeletal muscle mass, reduce adiposity, and improve glucose metabolism in patients with sarcopenia and other metabolic disorders, supporting their pleiotropic metabolic effects. However, the mechanistic role and therapeutic implications of myostatin modulation in the specific context of coexisting cirrhosis and sarcopenia remain incompletely understood. This review summarises current knowledge on myokine-mediated regulation of muscle metabolism in cirrhosis, with a focus on the myostatin–mTOR axis. We critically evaluate existing and emerging myostatin-targeting approaches, including pharmacological inhibition and gene-based therapies, as potential strategies to restore muscle mass and function. Finally, we outline future directions for translational research aimed at modulating myostatin signalling in cirrhosis-associated sarcopenia, emphasising the need to elucidate its systemic metabolic effects to identify novel therapeutic targets and improve clinical outcomes in this population.

## 1. Introduction

Liver cirrhosis represents the final stage of chronic liver disease, characterised by progressive fibrosis with distortion of tissue architecture and the formation of regenerative nodules [1,2]. The most common aetiologies of cirrhosis include metabolic dysfunction-associated steatohepatitis (MASH), alcohol-related liver disease (ALD), and chronic viral hepatitis B and C (HBV and HCV), with autoimmune and genetic disorders contributing less frequently [2,3]. Cirrhosis places a substantial burden on society and healthcare through premature mortality and high morbidity; decompensated cirrhosis is characterised by recurrent hospitalisation and significant healthcare utilisation, and progressive deterioration in quality of life [2,3,4]. The prevalence of cirrhosis has increased globally, with at least one million deaths per year [4]. The increase is driven by the rising prevalence of obesity and metabolic syndrome (including MASH) [5,6], while alcohol-related advanced liver disease remains important. Cirrhosis develops as a consequence of persistent hepatocellular injury arising from metabolic, toxic, viral, immune-mediated or cholestatic insults [7]. Cellular injury promotes chronic inflammation and immune activation, with subsequent activation of hepatic stellate cells and increased extracellular matrix deposition [7]. When matrix production exceeds degradation, progressive hepatic fibrosis develops. With ongoing injury, hepatic architecture becomes increasingly distorted, characterised by bridging fibrosis, regenerative hepatocyte nodules and abnormal vascular remodelling, resulting in increased resistance to portal blood flow and the development of portal hypertension. The haemodynamic consequences of portal hypertension are further amplified by splanchnic vasodilatation and activation of the renin–angiotensin–aldosterone system, sympathetic nervous system and vasopressin pathways, contributing to the development of ascites, varices and hepatorenal syndrome. Concurrently, loss of functional hepatocyte mass and portosystemic shunting impair hepatic synthetic and metabolic function, resulting in manifestations including hypoalbuminaemia, coagulopathy, jaundice and hepatic encephalopathy. The initially asymptomatic compensated phase of cirrhosis may progress to decompensation, characterised by the development of complications such as ascites, portal hypertensive bleeding or hepatic encephalopathy. Decompensated cirrhosis is associated with substantially impaired quality of life, recurrent hospitalisation and high mortality [4,7].

Reduced skeletal muscle mass and function (sarcopenia) is a common and clinically important finding in individuals with cirrhosis, and is influenced by various metabolic, hormonal, nutritional and inflammatory factors [1,8,9]. The reported prevalence of sarcopenia in cirrhosis varies according to the diagnostic criteria applied and the populations studied; however, it is estimated to affect approximately 30–70% of patients, with prevalence increasing in those with more advanced liver disease [8,9,10]. Sarcopenia has been identified as an independent predictor of mortality, conferring an approximately two-fold increase in the risk of death among patients with decompensated cirrhosis, independent of conventional measures of disease severity such as the Model for End-Stage Liver Disease (MELD) score [10,11]. Moreover, sarcopenia has been linked to a higher incidence of ascites, hepatic encephalopathy, and increased susceptibility to serious infections [12,13,14].

Age-related sarcopenia is characterised by the progressive loss of skeletal muscle mass accompanied by reduced muscle strength and/or impaired physical performance [9,13,14]. Sarcopenia in cirrhosis may exhibit distinct clinical and biological characteristics, with recent studies identifying potential cirrhosis-associated phenotypes and genetic or molecular markers [9,10,11,15,16]. However, a universally accepted cirrhosis-specific diagnostic phenotype has not yet been established. Unlike age-related sarcopenia, loss of skeletal muscle in cirrhosis may occur despite relatively preserved fat mass and body mass index (BMI), potentially obscuring clinically significant muscle depletion and functional impairment [9,10,15,16]. Muscle quality may also be adversely affected by myosteatosis, characterised by increased fat infiltration within skeletal muscle [17]. Although distinct from sarcopenia, myosteatosis frequently coexists with muscle depletion and is associated with metabolic dysfunction and adverse clinical outcomes, highlighting the importance of assessing muscle quality as well as muscle quantity in cirrhosis [17,18]. Both sarcopenia and myosteatosis are associated with poorer outcomes and may represent potentially modifiable contributors to disease progression. Importantly, sarcopenia should also be distinguished from muscle wasting occurring in other clinical settings, including inherited neuromuscular disorders and cancer-associated cachexia [13,16,19].

Key molecular drivers of muscle weakness (a sign of sarcopenia) include altered protein metabolism [20], hormonal imbalances [21], and a dysregulated myokine network [22]. Among the myokines implicated in sarcopenia associated with cirrhosis, myostatin has received particular attention because of its inhibitory effects on skeletal muscle growth [23]. Several studies have reported altered, and frequently elevated, circulating myostatin concentrations in patients with sarcopenia and cirrhosis [13,15,24], although the role of myostatin warrants further investigation [25]. The relationship between circulating myostatin, muscle wasting and the severity of liver disease remains incompletely understood, with conflicting findings reported in advanced liver disease [25]. A recent meta-analysis of ten clinical studies (2019–2024) indicated that myostatin is a fair biomarker of sarcopenia [26]. The physiological effect of myostatin is exerted through several pathways involving androgen receptor (AR) and mTOR (mechanistic target of rapamycin) signalling [19]. Recent therapeutic advances have led to the development of novel agents that target myostatin signalling pathways [13,19,27]. Several clinical trials are currently evaluating the efficacy and safety of myostatin inhibitors as potential therapies for sarcopenia. In studies of sarcopenic populations, myostatin inhibition has been associated with increased skeletal muscle mass, reduced adiposity, and improved glucose homeostasis, suggesting that modulation of myostatin exerts pleiotropic metabolic effects [27]. However, the role of myostatin inhibition in patients with cirrhosis-associated sarcopenia remains incompletely understood, and the underlying mechanisms require further investigation.

This review describes recent findings on the role of myostatin signalling in the pathogenesis of sarcopenia and examines the current evidence supporting myostatin-targeted therapies. Additionally, we discuss emerging therapeutic strategies and future research directions, including the potential application of genetic approaches to modulate myostatin activity as a strategy to ameliorate sarcopenia in patients with cirrhosis. This narrative review analysed recently published (the past decade) original articles and systematic reviews indexed in PubMed, the Cochrane Library, and a relevant clinical trial database (ClinicalTrials.gov). The search keywords included “sarcopenia”, “muscle”, “cirrhosis”, “liver”, and “myostatin” (separately and/or combined).

## 2. Assessment of Muscle Status in Clinical Practice: The Need to Unify and Consolidate Diagnostic Methods

In evaluating the impact of sarcopenia in cirrhosis, an appreciation of methods used to diagnose sarcopenia and their heterogeneity is important. Accurate and reproducible assessment of muscle mass and function is essential to identify patients at risk and monitor responses to therapy. Substantial heterogeneity exists in the methods used to diagnose and monitor sarcopenia [6,8,9,10,15,16]. This heterogeneity impacts efforts to summarise and compare different studies in the field [19,28]. Some diagnostic approaches rely exclusively on measures of muscle mass, such as the computed tomography (CT)-derived skeletal muscle index (SMI), calculated as the cross-sectional skeletal muscle area at the third lumbar vertebral level normalised for height squared [28,29,30,31]. The SMI has been shown to predict mortality in patients with cirrhosis [13]. Other anthropometric measures (more related to body composition) include bioelectrical impedance analysis, impedance plethysmography, and dual-energy X-ray absorptiometry [28]. In contrast, some approaches focus primarily on measures of physical performance or muscle function, including gait speed assessments [32], handgrip strength [30], and the Liver Frailty Index [8,9,29,33,34]. The diagnostic criteria, proposed by the Japan Society of Hepatology, incorporate both muscle strength and muscle mass, combining handgrip strength testing with assessment of muscle quantity using CT imaging or bioelectrical impedance analysis. These distinctions are clinically important, as measures such as the SMI quantify muscle mass but do not evaluate muscle function/quality. Assessments of muscle that solely use measures of muscle mass do not provide information on muscle weakness or reduced muscle function, nor do they take into account pathological changes such as myosteatosis, the abnormal accumulation of intramuscular fat, which affects muscle quality but is not associated with reduced muscle mass [6]. Therefore, muscle quality provides complementary information about muscle functional performance [35,36]. However, it remains poorly addressed or measured inconsistently in patients with sarcopenia (or with sarcopenic obesity).

Differences in diagnostic criteria also influence the identification of sarcopenia risk factors in clinical practice. Studies defining sarcopenia solely on the basis of reduced muscle mass, particularly using the SMI, have reported associations with male sex and alcohol-related cirrhosis [8,12,36]. In contrast, when diagnostic criteria incorporate measures of muscle function, such as handgrip strength or gait speed, these associations are attenuated or absent, and metabolic dysfunction-associated steatohepatitis (MASH)-related cirrhosis emerges as a more prominent risk factor [6,18]. There is no established and validated genetic signature for sarcopenia in cirrhosis. The field is still emerging, and most available evidence comes from indirect genetic approaches, transcriptomics in general sarcopenia, or studies in related liver diseases (e.g., MASH) rather than cirrhosis-specific cohorts [13,16,19]. It is likely that diagnosis of sarcopenia is best made using a combination of measures of muscle mass and function, and with criteria of severity [8]. Because of the different phenotypes (reduced muscle mass, muscle fat infiltration, reduced muscle strength), there may be differences in underlying pathophysiological mechanisms [37,38,39]. Markers of different sarcopenia phenotypes or of genetic predisposition to sarcopenia in cirrhosis may be helpful in clinical practice [14,15]. For instance, it remains unknown whether the severity of sarcopenia and loss of muscle function are associated with the stage of cirrhosis (compensated/decompensated) and which molecular mechanisms mediate the muscle–liver crosstalk [17,18,19,38].

## 3. Mechanisms of Muscle Loss in Cirrhosis: Convergence on Myokine Myostatin Signalling

**Figure 1:**
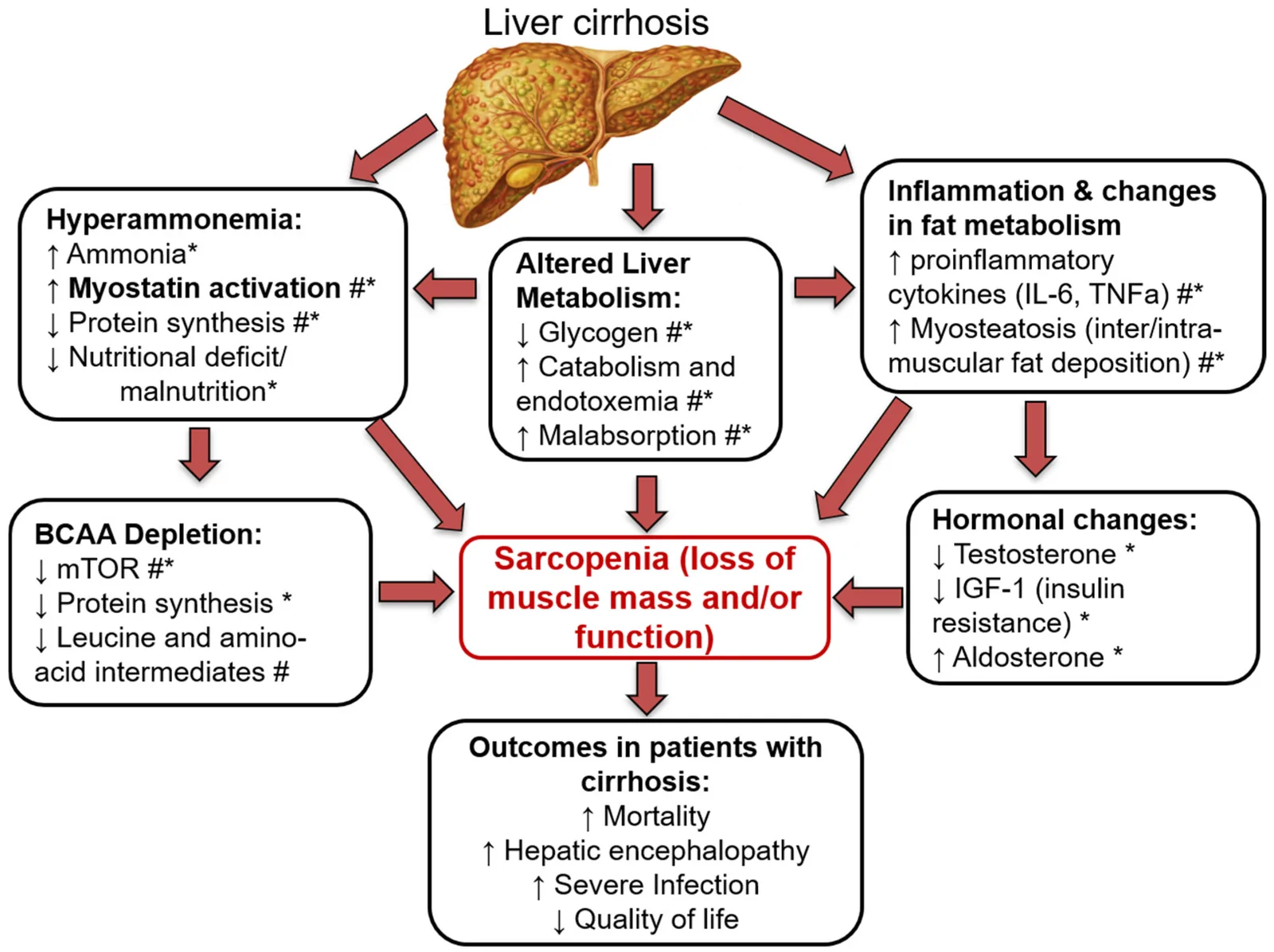
Sarcopenia in cirrhosis: major contributing mechanisms. Hyperammonaemia, protein catabolism, and branched-chain amino acid (BCAA) depletion contribute to the development of nutritional deficits, which lead to reduced muscle mass. Myostatin contributes to sarcopenia via the phosphoinositide 3-kinase (PI3K)/Akt/mammalian target of rapamycin (mTOR) signalling pathway, leading to impaired muscle growth and reduced functionality. Animal studies are indicated by #. Pathways and findings observed and/or confirmed in human studies are indicated by *. Lowered testosterone and insulin-like growth factor-1 (IGF-1) also contribute to the loss of skeletal muscle mass. The systemic pro-inflammatory status triggered by the liver disease and the increased intestinal permeability promote the pathogenesis of cirrhosis-associated sarcopenia.

The pathogenesis of sarcopenia (in both ageing and in cirrhosis) is complex and multifactorial, involving metabolic [9,10,13,20], nutritional [28,40,41,42,43], inflammatory [19,33,44], and hormonal (mostly steroid hormones [45,46,47] and myokines [23,28,30,31]) pathways. In patients with cirrhosis, liver metabolic dysfunction may lead to increased muscle protein degradation/autophagy, contributing to loss of muscle mass [12,13], which, in turn, is associated with a significantly worse prognosis [36,38,39]. Sarcopenia develops because of impaired muscle protein homeostasis, characterised by reduced muscle protein synthesis, increased proteolysis, and diminished regenerative capacity. These processes are driven by the metabolic derangements associated with chronic liver disease and contribute to the progressive loss of muscle mass and function. The associated mechanisms of sarcopenia and liver cirrhosis are presented in Figure 1.

Hyperammonaemia is considered a hallmark of cirrhosis and plays a central role in the development of sarcopenia [40,41]. Elevated plasma ammonia has been shown to impair muscle protein synthesis and promote muscle breakdown through activation of myostatin and other catabolic pathways [40,41]. In parallel, the expression of anabolic myokines, including irisin and interleukin-15, is reduced, further disrupting the balance between muscle protein synthesis and degradation [48,49]. Additionally, skeletal muscle acts as an alternative site for ammonia detoxification, further increasing metabolic stress on muscle tissue. Loss of muscle mass reduces this compensatory mechanism, contributing to elevated ammonia levels and neurotoxicity [20]. The relationship between sarcopenia and progression of hepatic encephalopathy is particularly important, highlighting the role of skeletal muscle in brain health [20,39], although further clinical studies are warranted to confirm the relationship.

Hormonal imbalances, particularly hypogonadism and low testosterone levels in men, contribute to sarcopenia. Testosterone plays a key role in maintaining muscle mass by stimulating protein synthesis and inhibiting protein breakdown. Low testosterone levels are common in cirrhosis and are associated with increased mortality [30,32,33,47]. The androgen signalling network helps to maintain muscle tissue balance at multiple levels, including regulation of myokine synthesis. Testosterone activates anabolic (protein synthesis) signalling via binding to the androgen receptor (AR) and its downstream targets, including the PI3K–Akt–mTOR pathway [50]. AR activation leads to inhibition of myostatin gene transcription and reduced SMAD signalling activity [45,51]. Accordingly, exogenous androgen administration has been shown to reduce myostatin mRNA expression in skeletal muscle (both human and animal studies) [21,47,52]. Recent data suggests that the synthesis of myokines is the key steroid hormone target in the metabolic regulation of muscle function [22]. For instance, testosterone (androgens) can counteract muscle loss through the blockade of myostatin transcription [53,54]. Accordingly, working through myostatin regulation, testosterone reduced sarcopenia in older adults [46].

A commonly observed sex difference is a roughly 2:1 male-to-female ratio among people with cirrhosis, although the exact ratio varies substantially by country, age, and underlying cause [55]. The higher global prevalence of cirrhosis in men is largely attributed to a combination of different intrinsic and extrinsic factors, such as alcohol consumption and viral hepatitis [55]. Biological sex differences in health behaviours and hormonal, metabolic (e.g., MASLD), and immune pathways additionally influence the development and progression of cirrhosis [56]. Notably, the sex-specific difference in the impact of sarcopenia in cirrhosis remains poorly investigated. The prevalence of sarcopenia in females with cirrhosis is generally lower [57], although the associated lower muscle mass and decreased muscle function were shown to contribute to mortality in women as well [11]. The reported differences may be affected by the methods used to diagnose sarcopenia and by the aetiology of cirrhosis. Studies that define sarcopenia solely based on measures of muscle mass report a male preponderance of sarcopenia, although the association with sex appears to diminish when definitions of sarcopenia incorporate a measure of muscle strength. These findings suggest that male testosterone status may have a role in sarcopenia in males with cirrhosis [57]. In contrast to men, low subcutaneous adipose tissue in women with cirrhosis may be associated with mortality [11]. Another study concluded that the contributors to frailty may differ between sexes in cirrhosis [58]. A recent review noted that, out of 35 studies that used sex-specific definitions to diagnose sarcopenia, 29 manuscripts did not stratify their outcomes by sex [57], suggesting a need to present sex-specific analyses of sarcopenia in patients with cirrhosis.

A major contributor to sarcopenia is increased skeletal muscle proteolysis, including autophagy-related protein degradation, which is influenced by cellular energy (glucose) supply [28,41]. Patients with cirrhosis exhibit a state of accelerated starvation due to reduced hepatic glycogen stores. As a result, the body relies increasingly on gluconeogenesis during relatively short periods of fasting, leading to increased muscle protein breakdown [41]. The metabolic shift in the diseased liver contributes significantly to muscle wasting. Consequently, nutritional optimisation has been suggested as a strategy for managing sarcopenia [59]. Frequent meals and late evening snacks have been shown to reduce catabolism during fasting periods [13,60].

Adequate protein intake is also vital to support muscle synthesis. Branched-chain amino acid (BCAA) supplementation is essential for protein synthesis and plays a role in regulating muscle metabolism [28,40]. In cirrhosis, increased utilisation of BCAAs for ammonia detoxification leads to their depletion, impairing protein synthesis and promoting muscle loss [40]. BCAA supplementation has been shown to influence mTOR signalling and reduce autophagy in cirrhotic muscles [42]. BCAA supplementation improves muscle mass and clinical outcomes, but evidence is heterogeneous, and benefits are often modest and context-dependent [40,43]. The strongest evidence for BCAA effects relates to hepatic encephalopathy, not necessarily robust reversal of sarcopenia [61]. Randomised controlled trials have demonstrated improvements in nitrogen balance, hepatic encephalopathy, and event-free survival [62,63]. BCAA supplementation increased muscle mass [63] but did not consistently influence the level of myostatin and other biomarkers of muscle activity [64]. No clinically meaningful improvement in skeletal muscle function was reported in one recent trial with this supplement [62], suggesting that BCAAs should be tested as a component of a more complex approach to target sarcopenia.

BCAA signalling and myokine synthesis (myostatin expression level) may be indirectly linked. It has been demonstrated in vitro that excess leucine (one of the BCAAs) may influence myostatin signalling and promote glucose sequestration and lipid accumulation in muscle cells (myosteatosis) [65], a condition associated with MASH-linked cirrhosis. In patients with MASH, chronic inflammation and some pro-inflammatory cytokines (such as interleukin-6 (IL-6)) contribute to muscle loss and myosteatosis through activation of catabolic pathways and inhibition of anabolic signalling [49,66]. Myosteatosis further exacerbates metabolic dysfunction and is associated with adverse outcomes [17,18]. Notably, BCAA signalling in myosteatosis is far less investigated, although the regulatory effects of BCAAs on the mTOR pathway (an upstream regulator of myostatin) were reported [67,68,69]. The interactions between BCAAs and liver function are far more complex and may be mediated by the gut–liver axis, involving a multicomponent system [37,69,70].

Complications of cirrhosis, such as abnormal protein metabolism (including BCAA depletion/signalling), reduced male testosterone status, and a pro-inflammatory environment, are all interconnected through myostatin/myokine signalling. Cirrhosis-related conditions seem to converge on the same core regulatory myokine networks that control muscle protein turnover (this will be discussed below).

## 4. Myostatin Signalling and Its Role in Sarcopenia

Skeletal muscle serves not only as the principal organ of locomotion but also plays a central role in energy production and homeostasis [71]. In addition to its mechanical functions, skeletal muscle synthesises and secretes a range of bioactive molecules known as myokines, which exert autocrine, paracrine, and endocrine effects. Consequently, skeletal muscle is increasingly recognised as an endocrine organ that contributes to the regulation of systemic metabolism [72]. Although the regulation of muscle growth is very complex, a therapy-focused approach requires a simplified understanding of the molecular mechanism that can be targeted by specific therapeutic agents. To present the manageable network, it has been described that skeletal muscle growth is regulated by antagonistic effects of insulin-like growth factor 1 (IGF-1, growth-promoting) and myostatin (growth-inhibiting). Myostatin expression in skeletal muscle varies, reflecting the stage of muscle growth or wasting [13,22,23].

A member of the transforming growth factor-β (TGF-β) superfamily, myostatin is a well-established negative regulator of skeletal muscle growth [73,74]. Following transcription of the MSTN gene, myostatin is initially synthesised as a precursor protein, pro-myostatin [75], which is subsequently cleaved to form latent myostatin (inactive). Following further proteolytic processing during maturation, mature and active myostatin can bind to the activin type II receptor B (ActRIIB) [75]. Therefore, several agents have been developed to target various forms of myostatin and its downstream receptors.

Myostatin, the activins, and growth differentiation factor 11 (GDF11) (members of the TGF-β superfamily [76]) all bind ActRIIA/ActRIIB and Alk4/Alk5 (activin) receptor complexes, trigger SMAD2/3 signalling and induce muscle atrophy [77]. This process is also marked by the inhibition of IGF-1-induced myoblast proliferation. Myostatin is an established inhibitor of the protein kinase B (Akt)/mTOR/p70S6K signalling pathway involved in protein synthesis. The pathway regulates both myoblast (muscle progenitor cells) differentiation and myotube hypertrophy [78,79]. Other muscle-wasting factors, including IL-6 and glucocorticoids, can also activate the SMAD2/3 signalling pathway, resembling the effects of myostatin [74].

Myostatin has been shown to blunt leucine-induced mTOR activation, interfere with nutrient sensing pathways, and affect amino acid signalling [78]. Even with adequate BCAA intake, high myostatin may be able to reduce the anabolic response [42], suggesting that myostatin and its receptor network may represent promising clinical targets in sarcopenia. Myostatin inhibition may counteract muscle wasting in cirrhosis as the expression of this myokine is elevated in catabolic states, including chronic liver diseases [23,27,28,30]. Genetic and pharmacological inhibition of myostatin signalling has been reported to increase skeletal muscle hypertrophy across multiple preclinical models and clinical studies, supporting its role as a therapeutic target [74,80]. However, the role of myostatin as a negative regulator of muscle function and as an associated contributor to cirrhosis-related complications requires further confirmation in human studies.

## 5. Convergence of Growth-Regulating Signalling on the mTOR Pathway in Muscle Cells

The mTOR pathway plays a central role in protein synthesis. Pharmacological modulation of this pathway may enhance anabolic signalling in skeletal muscle, although careful regulation is required due to potential adverse effects [54,78]. Both BCAAs and the androgen signalling pathway (growth-promoting signalling systems in muscles) activate muscle protein synthesis via the Akt/mTOR pathway. BCAAs, particularly leucine, activate mTORC1 and stimulate protein synthesis [81,82]. BCAA depletion in catabolic states (e.g., cirrhosis) is associated with reduced mTOR activity and reduced protein synthesis, leading to consequent muscle wasting. Increased expression of myostatin may amplify this effect. Myostatin may further reduce amino acid uptake, impair mitochondrial function, and promote catabolism (potentially exerting a synergistic effect in muscle loss) [40,83].

Although testosterone regulates amino acid metabolism at multiple levels, a major downstream effector of this hormone is the PI3K/Akt/mTOR network [84]. Therefore, testosterone replacement therapy was associated with increased amino acid uptake into muscle, improved protein synthesis efficiency, enhanced satellite cell activity, and more efficient utilisation of BCAAs [85]. Thus, BCAAs and testosterone are both anabolic (mTOR-activating) factors, while myostatin has catabolic effects, including inhibition of mTOR-signalling and stimulation of FOXO-mediated proteolysis [78,86]. It is plausible that in patients with cirrhosis, these three factors create a self-reinforcing catabolic cycle (pathological loop): when protein synthesis is impaired and testosterone levels are reduced, myostatin is up-regulated (and/or activated), leading to inefficient BCAA utilisation, inhibition of Akt/mTOR signalling, and further muscle wasting through FOXO-mediated proteolysis [86].

While mTOR is a central anabolic target with a complex signalling network of numerous metabolic regulators, it may not be an ideal direct pharmacological target [87,88]. In preclinical models, mTOR inhibition with rapamycin has been shown to extend lifespan and attenuate atherosclerosis [89] but has not consistently prevented muscle loss [90]. Strategies that activate mTOR, such as leucine supplementation and/or exercise, have improved muscle conditions but are not always sufficient as a standalone intervention [91]. This creates a therapeutic paradox: although mTOR is essential for muscle growth, direct pharmacological manipulation may be associated with undesirable systemic effects. Selected clinical approaches that target mTOR are presented in Table 1.

Accordingly, most successful approaches to preserving muscle mass and function include indirect mTOR modulators, including BCAAs/leucine and nutrient-sensing pathways [82], the testosterone/AR/Akt signalling pathway [84], and resistance exercise (mechano-sensing pathway) [35]. These methods have been tested separately and have not consistently achieved the desired outcome, although some beneficial effects have been reported. Moreover, one of the main clinical translation challenges is the lack of approved drugs specifically targeting mTOR for the treatment of sarcopenia. Therapeutic approaches targeting sarcopenia are discussed below.

## 6. Traditional Interventions for Sarcopenia

Because of its significant clinical consequences, pharmacological therapies that treat sarcopenia in cirrhosis represent an area of considerable interest. Most current interventions aim to modulate the underlying metabolic and hormonal imbalances. Exercise and nutritional therapy remain the cornerstone of sarcopenia management [31]. Resistance training is known to stimulate muscle protein synthesis via mTOR signalling, while adequate protein intake supports anabolic responses [99]. Resistance training has been shown to improve muscle mass and function in patients with cirrhosis, although clinical data is limited [63,100]. Future pharmacological strategies should therefore be designed as adjuncts to lifestyle interventions, rather than replacing them, to maximise therapeutic benefit. Despite these demonstrated benefits, lifestyle interventions, including exercise, are difficult to implement and adhere to in ageing populations and low-income socio-economic groups [101]. There are many impediments to overcome, including intrapersonal, interpersonal, and environmental barriers [100,101].

Hyperammonaemia and testosterone deficiency play central roles in the pathogenesis of sarcopenia. Consequently, testosterone replacement has been evaluated in randomised trials, which reported that additional testosterone can increase lean body mass and improve muscle strength [21,47,53]. Testosterone also improves insulin sensitivity and muscle glucose uptake [102]. Therefore, these pathways are interconnected through energy metabolism and the liver–muscle axis [103]. Selective androgen receptor modulators (SARMs; e.g., enobosarm) are under investigation for their anabolic effects in sarcopenia [104]. However, SARMs use may be limited by potential adverse effects, including cardiovascular risk and concerns regarding prostate-related adverse effects. Careful patient selection is therefore required. Testosterone therapy may benefit hypogonadal men [21]. Ammonia-lowering agents were also shown to support muscle preservation by reducing catabolic stress [28,30,41]. Therapies such as lactulose and rifaximin reduce systemic ammonia levels and may indirectly improve muscle metabolism. While preclinical and observational studies suggest benefit, robust clinical data demonstrating reversal of sarcopenia are lacking. These therapies represent promising therapeutic approaches but require further validation in larger clinical trials.

Given these barriers and the limited efficacy of existing interventions, emerging pharmacological approaches are focused on key molecular pathways involved in muscle wasting, including the myostatin pathway (Table 2). Pharmacological and gene-modulating methods have been used to inhibit myostatin expression and signalling. Both approaches have shown promising outcomes in early-phase studies by promoting muscle growth, although data in cirrhosis remains limited. Recent advances in myostatin inhibition and outcomes of clinical studies in sarcopenia are described below.

## 7. Myostatin Inhibition and Translational Therapeutics for Sarcopenia

### 7.1. Gene-Targeting Therapies for Myostatin

#### 7.1.1. CRISPR Gene Editing

To date, gene-targeting therapies for myostatin are still largely experimental. The tested agents that regulate myostatin expression are listed in Table 2. One of the most direct approaches is CRISPR (clustered, regularly interspaced, short palindromic repeats)/Cas9-mediated knockout of the myostatin gene (MSTN). CRISPR systems can introduce loss-of-function mutations in the myostatin gene [105]. This leads to increased muscle mass and hypertrophy, mimicking naturally occurring myostatin mutations seen in “double-muscle” phenotypes [106]. CRISPR-mediated myostatin disruption has been demonstrated in animal models, resulting in increased skeletal muscle mass and strength [107] and improved myogenic differentiation [105]. Instead of simple DNA cutting, CRISPR-based approaches can be used in several forms, including CRISPRi (interference), which suppresses myostatin transcription, or CRISPRa (activation), which can enhance inhibitors of myostatin pathways. CRISPR interference systems have been used in experimental models to repress myostatin gene expression without permanent genome editing [108]. To date, only animal and cell culture-based models have been tested. No MSTN gene-targeting therapy is currently approved for clinical use, partly because of concerns regarding the safety and potential irreversibility of genome editing. The long-term reversibility of the resulting phenotyping effects remains uncertain.

#### 7.1.2. RNA Interference (RNAi) and Antisense Oligonucleotide (ASO) Therapies

RNAi, particularly small interfering RNA (siRNA)-based therapies, have been used to reduce myostatin mRNA expression and decrease protein synthesis at the post-transcriptional level. siRNA-targeting myostatin in preclinical models has led to increased muscle fibre size, improved muscle regeneration, and enhanced functional performance [110,111]. However, effects were transient and required repeated dosing. Similarly, antisense oligonucleotides (ASOs) are short synthetic nucleic acids designed to bind myostatin mRNA and promote degradation or block translation. In contrast to siRNAs, ASOs are single-stranded RNAs or DNAs with about 15–25 nucleotides. ASOs can target pre-mRNA or mRNA and can be localised to both the nucleus and cytoplasm [126]. An ASO acts directly on its target, whereas a single siRNA-loaded RNA-induced silencing complex (RISC) can catalytically cleave multiple mRNA molecules [127].

Although ASOs offer greater design simplicity, they may have lower potency and specificity than siRNAs. However, ASOs can regulate not only transcription of a specific gene, but also RNA processing, splicing, and mRNA stability. For instance, an ASO binds myostatin pre-mRNA and promotes its degradation or alters splicing [112,118], while siRNA recruits RISC to cleave myostatin mRNA [110,111]. ASO-mediated myostatin suppression has been shown to increase muscle mass and improve muscle strength in mouse models [118]. A recently developed myostatin-specific ASO was reported to decrease myostatin protein levels in rhabdomyosarcoma cells [112]. Notably, ASOs can modulate gene expression through mechanisms that include RNA degradation, translational inhibition, and alteration of RNA processing or splicing [126,127]. This mechanistic versatility makes ASOs attractive. Many modern ASOs are chemically modified, highly protein-bound molecules that undergo tissue uptake and intracellular metabolism, rather than being primarily metabolised by hepatic cytochrome p450 (CYP) enzymes. Suggestively, hepatic impairment in cirrhosis may have less impact on ASO metabolism but may influence the agent uptake and distribution [128]. Importantly, the ASO models described above are designed to target muscle cells and, therefore, liver function may be less directly relevant to the pharmacodynamic effects when the target tissue is skeletal muscle.

#### 7.1.3. Gene Therapy Using Adeno-Associated Virus (AAV) Vectors and Non-Coding RNAs

AAV vectors can be used to deliver genetic constructs encoding dominant-negative myostatin, myostatin-binding proteins, or follistatin (a natural myostatin inhibitor). AAV vectors include engineered DNA expression cassettes packaged inside an AAV. The vectors can be delivered into cells to induce long-term expression of a therapeutic gene. AAV vectors are designed to deliver a therapeutic gene as episomal DNA, rather than to permanently edit or integrate the host genome. Unlike CRISPR-based genome editing, AAV-mediated approaches generally do not directly edit the MSTN gene [126]. Instead, they deliver genes that produce proteins capable of blocking the myostatin signalling pathway [113,114]. In spinal muscular atrophy models, the AAV-mediated expression of myostatin propeptide has been investigated and shown to have beneficial effects on muscle mass and sensory neural circuits [111,115,116]. To achieve sustained suppression of myostatin signalling, the follistatin genes were introduced using AAV vectors. AAV-follistatin gene therapy with AAV1:FS344 (the construct containing the follistatin-344 (FS344) transgene, which encodes follistatin isoform FS315) has been shown to increase muscle mass and improve muscle function in preclinical and early clinical studies [74,113,116]. FS315 can be released from muscles and binds extracellular myostatin [74]. There are other AAV vector constructs, including AVV-dnMSTN (dominant-negative myostatin constructs) [129] and AAV-ActRIIB (encoding a soluble activin receptor [130]), which were successfully tested in mouse models.

Enhanced muscle regeneration and improved muscle strength were reported in models of muscular dystrophy [113,116] and in patients with myositis [116]. This is one of the most advanced gene-based approaches and has entered early-phase human studies in muscular dystrophy. Follistatin-targeting agent (rAAV1.CNV.huFOllistatin344; FST-344) is currently in clinical trials (https://clinicaltrials.gov/study/NCT02354781; accessed on 25 June 2026). The main limitation of this method is a side effect of immune responses to viral vectors.

Epigenetic strategies that aim to regulate myostatin expression include histone modification, DNA methylation, and non-coding RNAs (such as microRNA (miR)). In contrast to siRNAs (exogenous molecular constructs, gene-specific silencers), miRs, which primarily regulate gene expression post-transcriptionally, are naturally produced by the cell and regulate normal physiological processes. The main difference from siRNA is partial complementarity: a single miR can bind many different mRNAs. Like ASOs, mature miRs are represented by single-stranded RNAs derived from a hairpin precursor and typically contain 21–23 nucleotides. Their main biological function is to fine-tune the expression of multiple genes, potentially hundreds, via translational repression and mRNA destabilisation [126]. Thus, miRs can ultimately reduce gene and protein expression, although changes in one miR can also indirectly influence the expression of other genes [117]. Several miRs (miR-1 [119], miR-206 [110], miR-29b [121], miR-542-5p [122], miR-486-5p [123], miR-23a, and miR-27a [124]) have been identified as regulators of muscle mass and function. Myostatin was also shown to regulate the expression of specific miRs, including miR-431 [125]. Some of these miRs have been reported to function as “atro-miRs” and promote muscular atrophy associated with the loss of muscle mass [124,131]. The injection of AAV-miR-23a/27a was tested in a mouse model of diabetes, preventing muscle wasting [124]. No miR-related interventions have yet been evaluated in clinical models.

The lack of clinical testing is associated with several key challenges of gene-based therapies, including challenges in skeletal muscle delivery and safety concerns. Efficient and targeted delivery remains a major barrier, compounded by concerns regarding off-target genetic effects and unknown long-term risks. Moreover, the effectiveness of AAV-mediated gene delivery may be reduced in tissues containing actively dividing cells in younger patients, as episomal AAV genomes can be diluted during cell division. The presence of actively dividing cells makes durable expression less likely. Cirrhosis may alter the pharmacokinetics, tissue distribution, cellular uptake, and safety profile of nucleic-acid and viral-vector therapies. The magnitude and direction of these effects depend strongly on the therapeutic platform, route of administration, target tissue and disease severity [132]. While hepatic dysfunction may complicate liver-directed delivery, it may have a more limited effect on therapies targeting extrahepatic tissues such as skeletal muscle.

In conclusion, CRISPR-based approaches offer the most permanent suppression of myostatin but are currently limited to preclinical research. CRISPRi- and miR-based epigenetic approaches represent emerging regulatory strategies rather than direct gene deletion. These approaches may offer greater reversibility than permanent genome editing, but their safety requires further investigation. RNAi and ASO therapies also provide reversible suppression but require repeated administration. AAV-mediated gene therapy (especially follistatin-based tactics) is the most clinically advanced gene-based approach with early human studies. However, data is limited. Future clinical studies should be focused on the specific cohort of patients with both cirrhosis and sarcopenia.

### 7.2. Myostatin Inhibitors for the Treatment of Muscle Wasting

Myostatin inhibition strategies include the use of ActRIIB ligand traps, myostatin-neutralising antibodies, and myostatin propeptides [133] (summarised in Table 3). Myostatin-neutralising antibodies and myostatin propeptides can provide relatively specific inhibition of myostatin signalling, whereas broader inhibition of the activin/myostatin pathway may produce additional effects. For instance, their off-target effects were found to be beneficial, including a reduction in age-associated degeneration [134,135]. For instance, treatment with myostatin inhibitors in mice was associated with reduced hyperlipidaemia and inflammation [136,137]. SRK-015 (apitegromab) is a highly selective monoclonal antibody (mAb) that binds latent myostatin and prevents its activation. It has been investigated in mice with sarcopenia (preclinical models) and clinically in patients with spinal muscular atrophy (SMA). Apitegromab demonstrated high specificity and was further approved for clinical trials (Phase II, spinal muscular atrophy) [138].

Early clinical trials of myostatin/activin pathway inhibitors included mAbs and ActRIIB antagonists. These trials demonstrated dose-dependent increases in lean muscle mass, but functional outcomes were variable [139,140]. For example, ActRIIB blockade with bimagrumab has been shown to increase lean body mass and reduce fat mass in humans [139,141]. Bimagrumab binds ActRIIA and ActRIIB and competitively prevents the binding and signalling of myostatin, activin A, and other ligands of these receptors [74,142]. However, improvements in muscle strength and functional mobility have not been consistently demonstrated across clinical trials with this agent [23]. The development of bimagrumab has been discontinued for several indications [23]. A recent study on common marmosets indicated that ACE-031, a soluble ActRIIB extracellular domain fused to human IgG1, increased lean body mass and muscle strength [143].

**Table 3:** Recent and ongoing clinical studies investigating myostatin and related pathway inhibition.

Agent	Target/Mechanism	Study Population	Phase	Key Findings	Status and Refs.
Trevogrumab + semaglutide *	Anti-myostatin (GDF-8) antibody	Obesity	Phase II COURAGE trial	Preserved ~50–80% of lean mass lost with GLP-1R therapy	Ongoing COURAGE trial NCT06299098
Trevogrumab ± garetosmab	Myostatin + activin A inhibition	Obesity	Phase II COURAGE trial	Improved body composition; reduced lean mass loss	Active trial (COURAGE NCT06299098) presented at EASL conference 2025
Apitegromab (SRK-015) + GLP-1R agonists	Myostatin activation inhibitor	Obesity/metabolic disease	Phase II/III TOPAZ and EMBRAZE trials	Investigating muscle preservation during weight loss	Recruiting/ongoing, NCT06445075 [144,145,146]
Bimagrumab (BYM338)	ActRIIB antagonist (blocks myostatin/activin)	Obesity/sarcopenia	Phase II	Improves lean mass; variable functional outcomes	New Phase II trials planned/ongoing (2025) NCT01669174; NCT01601600
Bimagrumab (BYM338) + tirzepatide	Activin type II receptor antagonist (blocks myostatin + activin A) Combination therapy	Sarcopenia, body myositis, Obesity + T2DM	Phase IIb RCT	↑ Lean body mass (~5–7%); improved body composition	Trial halted 2025 [75] or discontinued due to inconsistency
Apitegromab (SRK-015)	Selective myostatin activation inhibitor	Spinal muscular atrophy	Phase II/III	Improved motor function in neuromuscular disease	Late-stage development; regulatory evaluation ongoing; Not yet studied in cirrhosis/sarcopenia [146,147]
Landogrozumab LY2495655	Myostatin monoclonal antibody	sarcopenia/frailty, hip replacement	Phase II RCT	Improved muscle mass, reduced fat mass, some improvement in physical performance	Not approved; trial halted [148]
Taldefgrobep alfa (BMS-986089)	Anti-myostatin antibody and myostatin pathway inhibitor	Muscle wasting disorders	Phase III	Increased muscle mass signals; Safety and efficacy evaluation ongoing	Expected completion ~2025–2026 [80]
GYM329 (RO7204239)	Anti-myostatin antibody	Muscular disorders	Phase II	Investigational; improving muscle mass in early studies	Ongoing/early-phase [149]
Trevogrumab (REGN1033 Regeneron) (monotherapy)	Myostatin monoclonal antibody	Healthy adults/sarcopenia and obesity	Early phase testing	Muscle mass increase signals observed	Investigation in progress, NCT01910220 and NCT01963598, expanding indications [150]
Dual-blockade myostatin + activin inhibition	Dual pathway blockade	Obesity/metabolic disease	Phase II	Enhanced muscle preservation vs monotherapy.	Emerging 2024–2025 strategy [77]
Stamulumab (MYO-029)	Anti-myostatin monoclonal antibody	Adult muscular dystrophy	Phase II RCT	Well tolerated but no significant improvement in muscle strength or function	Recently confirmed lack of efficacy led to discontinuation [151]
Follistatin gene therapy (rAAV1.CMV. FS344)	Myostatin antagonist (binds ligands)	Muscle dystrophy	Early-phase testing	↑ Muscle mass	Limited clinical translation [113]
AMG-745 (peptibody from Amgen)	Myostatin pathway inhibitor, Fc-fusion protein	Cancer cachexia	Early-phase testing	↑ Muscle mass, ↓ fat mass; no improvement in muscle function	No clear functional benefit found; trial halted [152].

Abbreviations: Randomised controlled trial—RCT. * Semaglutide, a GLP-1R agonist, has been used for MASH treatment recently, although no improvement in liver fibrosis or steatosis has been noted [144,145].

Another myostatin-specific mAb, RG6237/GYM329 (emubrobart), was engineered with a sweeping mechanism that facilitates the clearance of myostatin from plasma and muscle tissue [153]. The agent is currently being evaluated in a Phase II clinical trial in combination with tirzepatide, a dual glucose-dependent insulinotropic polypeptide (GIP)/glucagon-like peptide-1 (GLP-1) receptor agonist (anti-diabetic agent), in adults with overweight or obesity and at least one weight-related comorbidity [154]. It remains to be confirmed whether the inhibition of myostatin (pathway) is sufficient to reverse sarcopenia, particularly in complex conditions such as cirrhosis. Considering that metabolic dysfunction, inflammation, and hyperammonaemia contribute to muscle loss, combined therapy seems more reliable for the treatment of sarcopenia in cirrhosis.

The discrepancy between increases in muscle mass and improvements in muscle function and neuromuscular integration remains a critical limitation of myostatin-targeted monotherapy. Over the last decade, therapeutic strategies for sarcopenia have increasingly shifted towards combination therapies. Combination therapies targeting multiple metabolic and anabolic pathways may be required to achieve clinically meaningful outcomes in sarcopenia and cirrhosis. While earlier studies used single myostatin inhibition, new approaches aim to evaluate combination regimens or agents with multitarget activity, such as simultaneous inhibition of myostatin/activin A signalling combined with incretin-based therapy (GLP-1R agonists) (Table 3). These approaches may enhance lean mass preservation, improve metabolic outcomes, and provide synergistic biological effects on muscle function.

Several clinical trials are in progress as Phase II and Phase III trials [155]. Notably, these trials have been conducted on obesity/TIIDM and neuromuscular disease (not liver cirrhosis). In the recent clinical trial NCT02944500 (http://clinicaltrials.gov/show/NCT02944500; accessed on 25 June 2026), liraglutide treatment was associated with suppression of circulating myostatin and changes in several proteins involved in metabolic and vascular remodelling pathways [156]. Recent interim results from the Phase II COURAGE trial (https://clinicaltrials.gov/show/NCT06299098; accessed on 25 June 2026) demonstrated that trevogrumab, alone or in combination with garetosmab, when added to semaglutide (GLP-1 receptor agonist), preserved more lean body mass and improved body composition during weight loss than semaglutide alone [144,155]. Trevogrumab combined with semaglutide preserved approximately 51% of the lean mass that would otherwise have been lost with semaglutide alone, while the combination of trevogrumab, garetosmab, and semaglutide preserved approximately 81%.

In diet-induced obese mice, SRK-439 (myostatin-specific mAb, binds ActRII) was evaluated together with metformin (common metabolic therapy). The approach demonstrated good preservation of lean mass during weight loss, enhanced fat loss while maintaining muscle tissue, and improved insulin sensitivity and energy metabolism [145]. Mechanistically, myostatin inhibition may counteract muscle catabolism during weight loss (caloric restriction) by promoting anabolic signalling (protein synthesis) and reducing muscle protein breakdown (proteolysis) [157]. Although muscle functional improvement remains inconsistent, these findings provide a mechanistic rationale for evaluating myostatin inhibition and GLP-1R agonism in cirrhosis-related sarcopenia.

Trevogrumab has also been evaluated in combination with garetosmab (an anti-activin A mAb), resulting in increased muscle volume [158]. These findings highlight that dual- or triple-pathway inhibition (myostatin/activin A ± incretin or other agent signalling) may represent a promising future strategy for preserving skeletal muscle mass. For instance, the Phase II COURAGE trial evaluated trevogrumab, with or without garetosmab, in combination with semaglutide in adults with obesity, and demonstrated preservation of lean mass during pharmacological weight loss [142]. Recent evidence from another Phase II trial demonstrated that combining semaglutide with trevogrumab, with or without an anti-activin A agent, significantly preserved lean mass during weight loss [156,159]. Landogrozumab (anti-myostatin mAb) was tested in older adults with sarcopenia and muscular atrophy [148]. The agent demonstrated improvements in some functional measures, including stair climbing performance [148], although the other study did not achieve its primary objective [160].

Aside from muscle mass, agents such as apitegromab (SRK-015) have been shown to improve muscle motor functions while demonstrating a good level of safety in early-phase trials [146]. Apitegromab has advanced into late-stage clinical development, with regulatory submissions underway, marking one of the first potential approvals in the myostatin inhibitor class. Although most trials have been conducted on patients with obesity/TIIDM and neuromuscular disease/muscular dystrophy [161], the findings are considered relevant to cirrhosis. Patients with cirrhosis may experience accelerated muscle wasting driven by hyperammonaemia, systemic inflammation, altered metabolism, and other disease-related factors [162]. Although these findings may provide a rationale for investigating myostatin inhibition in cirrhosis-related sarcopenia, their clinical relevance to patients with cirrhosis remains to be established.

In conclusion (Table 3), despite the increased lean body mass observed with myostatin pathway inhibition, clinical outcomes have been inconsistent, particularly with respect to muscle strength and physical performance. This discrepancy highlights the complex pathophysiology of sarcopenia in association with other pathologies, where metabolic, inflammatory, and hormonal factors extend beyond myostatin signalling alone [162]. Across several clinical trials (Table 3), myostatin pathway inhibition increased lean body mass or muscle volume; however, the magnitude of these changes varied between studies and did not consistently translate into functional improvements. Broader pathway inhibition, including simultaneous targeting of myostatin and activin A signalling (e.g., bimagrumab), may produce greater increase in muscle mass than selective myostatin inhibition in some experimental settings; however, comparative clinical evidence remains limited. Current clinical evidence is derived from studies of neuromuscular disorders, older adults with frailty/sarcopenia; cancer cachexia; and, more recently, obesity and pharmacological weight-loss populations. No large-scale randomised controlled trials have yet evaluated myostatin inhibitors specifically in patients with cirrhosis.

While myostatin inhibitors have demonstrated acceptable short-term safety profiles, long-term data remain limited. Potential concerns include off-target effects on cardiac and smooth muscle, potential endocrine disruption, and unknown long-term metabolic consequences. This represents a significant gap in the literature and a key priority for future research. Furthermore, heterogeneity in patient populations and outcome measures across clinical trials complicates interpretation and comparison of results. Pharmacological response may also differ across different groups (compensated vs. decompensated cirrhosis; MASH vs. ALD and others). Therefore, management of sarcopenia in cirrhosis requires a multifaceted approach targeting underlying mechanisms, including the myostatin pathway. Future trials should include functional endpoints (e.g., grip strength and gait speed), standardised measures of muscle quality (the consistently chosen marker, e.g., muscle attenuation or intramuscular fat), and long-term follow-up.

### 7.3. Pharmacological Targets for Combination Therapy with Myostatin Inhibitors

BCAA availability, testosterone signalling, and the myostatin/TGF-β pathway form an interconnected network that influences Akt/mTOR signalling [50,70,73,78,88]. There are several well-described molecular mechanisms linking myostatin and androgens/AR signalling. The androgen pathway is central to skeletal muscle regulation and is highly relevant to sarcopenia, cachexia, and liver disease-associated muscle wasting. Myostatin and testosterone regulate muscle mass through opposing yet interconnected pathways [99,163]. Myostatin activates SMAD2/3 signalling and can suppress Akt/mTOR, thereby promoting muscle protein catabolism. In contrast, testosterone-mediated AR signalling can stimulate anabolic pathways, including the Akt/mTOR network, and support muscle protein synthesis. This mechanistic interplay supports a rationale for combination therapies. Activation of anabolic pathways by testosterone may complement myostatin inhibition. Such a combination could theoretically enhance muscle protein synthesis while reducing muscle protein breakdown, providing a complementary anabolic- anticatabolic strategy.

Enobosarm (GTx-024; SARM), a selective AR modulator (SARM), is designed to activate AR in skeletal muscle [104]. The agent activates the PI3K-Akt-mTORc1 anabolic network and promotes muscle growth and maintenance [104]. Enobosarm has also demonstrated improvements in selected measures of physical function in clinical studies [164]. In combination with semaglutide (an anti-diabetic and weight-reducing drug), enobosarm has been investigated as a strategy to preserve lean body mass during pharmacological weight loss in the Phase IIb QUALITY trial [165]. However, to date, no clinical trials have evaluated the combination of a myostatin inhibitor and enobosarm in patients with cirrhosis.

The efficacy of testosterone supplementation or myostatin inhibition may be influenced by nutritional status, including inadequate BCAA and protein availability. Therefore, triple-axis intervention seems even more promising. Such a strategy could combine BCAA supplementation, testosterone optimisation in patients with confirmed deficiency, and myostatin inhibition. The treatment strategy could be individualised according to patient characteristics, nutritional status, hormonal imbalances, and disease severity. When used as a combination therapy, BCAAs may provide nutritional support for muscle protein synthesis, testosterone may enhance androgen-mediated anabolic signalling in patients with deficiency [84,166], and myostatin inhibition may reduce myostatin-mediated inhibitory signalling.

Moreover, future therapeutic strategies are likely to involve multitarget approaches, including ghrelin and appetite-modulating agents [155]. This approach is supported by observations that poor appetite and inadequate protein intake contribute to the development of sarcopenia in patients with cirrhosis [167]. One potential agent is Anamorelin (a ghrelin receptor agonist), which has been evaluated in a Phase III clinical trial involving patients with cancer cachexia [168]. Ghrelin is an orexigenic hormone that stimulates appetite. Ghrelin receptor agonists stimulate appetite and may enhance muscle anabolism indirectly by increasing energy/caloric intake and growth hormone secretion [88]. The role of myostatin in appetite regulation remains unclear. However, recent studies suggest that calorie-restricting diets may decrease myostatin expression [169]. In a recent randomised controlled study of 42 patients with cirrhosis, a vegetable- and dairy-protein-based diet was associated with lower increases in blood ammonia concentrations compared with the standard diet [170]. Although muscle function did not improve significantly compared with the standard diet, these findings support further investigation of dietary strategies for managing sarcopenia and hyperammonaemia in cirrhosis.

## 8. Future Research Priorities and Conclusions

Sarcopenia is a common and clinically significant complication of cirrhosis that is associated with adverse outcomes, including increased mortality, complications, and reduced quality of life. Its pathogenesis is multifactorial, involving metabolic, hormonal, and inflammatory mechanisms. Severe sarcopenia and reduced muscle regeneration are often marked by a poor response to nutritional intervention alone and require a multimodal approach. Importantly, sarcopenia is a potentially modifiable condition.

Myostatin inhibitors represent a major advancement in the pharmacological management of muscle wasting, with recent trials demonstrating their ability to preserve or increase lean body mass, particularly when used in combination with incretin-based therapies [146,147]. Myostatin inhibition represents a promising therapeutic strategy for sarcopenia; however, its clinical utility remains limited by inconsistent functional outcomes when used as monotherapy [144,145,156,157,158,159], although they are generally well tolerated [103]. Despite substantial advances in sarcopenia treatment with myostatin inhibitors, the absence of cirrhosis-specific trials remains a key limitation. Another important limitation is the lack of well-established circulating myostatin levels in large cohorts of patients with both sarcopenia and cirrhosis. Notably, different age groups and stages of cirrhosis should be included. The liver-related characteristics are particularly important, as one recent manuscript indicated conflicting data regarding lower myostatin levels in patients with liver failure [25]. To facilitate the translation of myostatin-targeted therapies into clinical practice, future research should prioritise cirrhosis-specific clinical trials that account for disease stage, including compensated and decompensated cirrhosis, as well as the heterogeneity of sarcopenic phenotypes [9]. Given the complex metabolic disturbances in cirrhosis, this population provides a valuable model in which to evaluate the potential synergistic effects of myostatin inhibition with other metabolism-directed therapies [83,171]. Assessment of muscle quantity and quality should be appropriately adapted to patients with cirrhosis, with consideration of cirrhosis-specific biomarkers. However, the biomarker field for this pathology remains at an early stage of development.

The development of biomarker-guided and broad-spectrum therapies represents a promising research strategy. Future studies should focus on identifying biomarkers that reflect activation of the myostatin signalling pathway and characterise metabolic function in skeletal muscle, thereby enabling improved patient selection and treatment monitoring. Emerging evidence suggests that the gut microbiome plays an important role in both skeletal muscle homeostasis and liver-related complications [172]. Recent randomised controlled trials have highlighted the potential of microbiome-directed therapies to influence muscle health while simultaneously reducing complications such as hepatic encephalopathy [173]. Future therapeutic strategies may benefit from integrating microbiome modulation with inhibition of myostatin signalling.

Beyond monoclonal antibodies and receptor antagonists, gene-targeting approaches represent an emerging strategy for the modulation of myostatin signalling. These interventions aim to achieve sustained or potentially permanent suppression of myostatin activity and are therefore attracting considerable interest in preclinical and early-phase clinical research. A recent RNA sequencing study of age-related sarcopenia demonstrated widespread alterations in gene expression and dysregulation of alternative splicing pathways in muscle cells [174]. Advances in transcriptomic profiling may identify promising biomarkers and therapeutic targets. For instance, overlapping molecular signatures between MASLD and sarcopenia were identified, revealing shared pathways involved in metabolism, inflammation, and tissue remodelling [175]. These findings suggest the existence of common biological mechanisms linking liver disease and skeletal muscle dysfunction. However, the relevance of these observations to cirrhosis-associated sarcopenia remains uncertain and requires further investigation. Integration of muscle and liver transcriptomic and proteomic data with circulating microRNA profiles may provide a more comprehensive understanding of the role of sarcopenia in cirrhosis. Importantly, candidate molecular signatures should be validated in independent cohorts to ensure reproducibility and clinical applicability.

Myostatin inhibition is a biologically rational strategy that may promote skeletal muscle regeneration and increase muscle mass. However, the translation of these effects into meaningful improvements in muscle quality and physical performance in cirrhosis remains uncertain. Cirrhosis-associated sarcopenia arises from a complex interplay of metabolic, inflammatory, hormonal, and hyperammonaemia-related mechanisms, involving modulation of myostatin signalling. Therapeutic strategies should be evaluated within multimodal frameworks that combine myostatin-targeted interventions with established approaches such as nutritional optimisation, exercise training, ammonia-lowering therapies, and correction of hormonal abnormalities. Well-designed, adequately powered clinical trials in patients with cirrhosis are required to define the efficacy, safety, and optimal role of myostatin inhibition in the management of cirrhosis-associated sarcopenia. By combining targeted modulation of myostatin signalling with comprehensive management of the underlying metabolic and systemic disturbances in cirrhosis, future therapies may more effectively improve muscle and functional status in this vulnerable population.

## Figures and Tables

**Table 1:** Selected mTOR network-targeted therapies and associated effects in muscles: summary of clinical and preclinical evidence.

Approach/Agent	Mechanism of Action	Model/Study Type	Key Findings	Limitations/Concerns	Refs.
Rapamycin (sirolimus)	Direct inhibition of mTORC1, reducing protein synthesis and modulating autophagy	Preclinical (ageing and muscle wasting models)	Extends lifespan and improves some age-related phenotypes in animals; chronic mTOR inhibition may impair anabolic responses and muscle maintenance depending on dose	Muscle atrophy risk; immunosuppression; not muscle-specific	[92]
Rapalogs (everolimus, temsirolimus)	Partial mTORC1 inhibition	Clinical (oncology, limited muscle studies)	mTOR inhibition associated with muscle loss as an adverse effect in kidney transplants/cancer patients	Not anabolic; adverse metabolic effects	[77,93].
mTOR activation via leucine/BCAAs	Leucine activates mTORC1 via Rag GTPases	Clinical and preclinical (sarcopenia, liver disease)	Improves muscle protein synthesis; used in cirrhosis-related sarcopenia	Effect diminished in insulin resistance and high myostatin states	[40,70,82]
β-hydroxy β-methylbutyrate (HMB)	Activates mTOR signalling and reduces proteolysis	Clinical trials (elderly, sarcopenia)	Increases lean body mass and reduces muscle breakdown	Effects modest; inconsistent functional improvement	[94]
Resistance exercise (mechanistic activation)	Activates mTOR via mechanical loading and Akt signalling	Clinical (gold standard intervention)	Strongest evidence for mTOR activation and muscle hypertrophy	Requires adherence; limited in frail patients	[95]
Testosterone therapy (TRT)	Activates Akt–mTOR pathway and suppresses FOXO-mediated degradation	Clinical trials (hypogonadism, sarcopenia)	Increases muscle mass and strength; enhances mTOR signalling	Cardiovascular risk; not universally recommended	[53,84]
Prospective mTOR activators (experimental compounds)	Direct or indirect activation of mTOR pathway	Preclinical	Enhance muscle protein synthesis and hypertrophy in models	Limited clinical translation; safety concerns	[88,96]
Metformin (indirect mTOR modulation)	Indirect inhibition of mTOR via AMPK activation	Clinical and preclinical	May improve metabolic health but can reduce anabolic signalling	Potential to regulate muscle function via direct impact on the mitochondrial intrinsic function in ageing	[97]
AMPK–mTOR axis modulators	AMPK activation inhibits mTOR signalling	Preclinical	Improves metabolic efficiency; may counteract anabolic pathways	Catabolic effect may worsen sarcopenia if excessive	[98]
Exercise + nutritional mTOR activation (combined therapy)	Synergistic activation of mTOR via mechanical + nutrient signalling	Clinical (elderly, sarcopenia)	Most effective strategy for maintaining muscle mass and function	Requires compliance; not pharmacological	[8]

Abbreviations: AMPK—AMP-activated protein kinase; mTOR—mechanistic/mammalian target of rapamycin.

**Table 2:** Promising gene-editing approaches to control myostatin (MSTN) expression and signalling.

Approach	Mechanism of Action	Example(s)	Stage	Key Findings	Limitations/Challenges	Refs.
CRISPR/Cas9 gene editing	Direct knockout or disruption of the MSTN gene at the DNA level	CRISPR-Cas9 MSTN knockout models	Preclinical (animal models)	Increased skeletal muscle mass and hypertrophy; “double-muscle” phenotype replicated	Off-target mutations; delivery challenges; ethical concerns; irreversibility	[105,106,107]
CRISPR interference (CRISPRi)	Transcriptional repression of MSTN without DNA cleavage	dCas9-KRAB systems targeting MSTN promoter	Preclinical	Reduced MSTN expression without permanent genome editing	Transient or partial effect; delivery efficiency	[108,109]
RNA interference (siRNA/shRNA)	Post-transcriptional silencing of MSTN mRNA	siRNA targeting MSTN	Preclinical	Increased muscle fibre size and improved regeneration in models	Short duration of action; repeated dosing required	[110]
Antisense oligonucleotides (ASOs)	Binding to MSTN mRNA to block translation or induce degradation	MSTN-targeting ASOs	Preclinical	Improved muscle mass and function in experimental models	Delivery to muscle; stability; cost	[111,112]
Viral vector-mediated gene therapy	Viral vector delivers genetic constructs that inhibit MSTN signalling	adeno-associated virus (AAV)-follistatin, AAV-dominant-negative MSTN	Preclinical/early clinical	Sustained increase in muscle mass and strength; long-term expression	Immune response; safety concerns; irreversibility	[113,114]
Follistatin gene therapy	Overexpression of follistatin (natural inhibitor of MSTN and activin A)	AAV-follistatin constructs	Preclinical/early clinical trials	Significant muscle hypertrophy; enhanced regeneration	Potential off-target endocrine effects; long-term safety unknown	[115,116]
MicroRNA-based regulation	Endogenous miRNAs suppress MSTN gene expression	miR-1, miR-206, other myo-miRs	Preclinical	Regulation of muscle differentiation and growth; modulation of MSTN signalling	Indirect effect; delivery challenges	[117,118,119,120,121,122,123,124,125]
Gene editing of regulatory pathways	Targeting upstream regulators of MSTN (enhancers, transcription factors)	Experimental CRISPR-based regulation of enhancers	Preclinical	Potential fine-tuning of MSTN expression	Limited evidence; early-stage research	[108]

Abbreviations: AAV—adeno-associated virus; CRISPR—clustered, regularly interspaced, short palindromic repeats; MSTN—myostatin.

## Data Availability

No new data were created or analysed in this study. Data sharing is not applicable to this article.

## Abbreviations

ActRIIA or ActRIIB	Activin receptors A or B
AAV	Adeno-associated virus
ASOs	Antisense oligonucleotides
ALD	Alcohol-related liver disease
AR	Androgen receptor
BCAAs	Branched-chain amino acids
CRISPR	Clustered, regularly interspaced, short palindromic repeats
HBV and HCV	Chronic viral hepatitis B and C
CT	Computed tomography
GLP-1	Glucagon-like peptide-1
GDF11	Growth differentiation factor 11
IL-6	Interleukin-6
IGF-1	Insulin-like growth factor 1
mTOR	Mechanistic target of rapamycin
MASH	Metabolic dysfunction-associated steatohepatitis
MASLD	Metabolic dysfunction-associated steatohepatitis liver disease
MELD	Model for End-Stage Liver Disease
mAb	Monoclonal antibodies
PI3K/Akt	Phosphoinositide 3-kinase
siRNA	RNA interference, RNAi or small interfering RNA
SARM	Selective androgen receptor modulator
SMAD	Signal transducer for TGF-β network/intracellular transcription factors
SMI	Skeletal muscle index
TGF-β	Transforming growth factor-β
TNFα	Tumour necrosis factor α
